# Lower Fetuin-A, Retinol Binding Protein 4 and Several Metabolites after Gastric Bypass Compared to Sleeve Gastrectomy in Patients with Type 2 Diabetes

**DOI:** 10.1371/journal.pone.0096489

**Published:** 2014-05-06

**Authors:** Mia Jüllig, Shelley Yip, Aimin Xu, Greg Smith, Martin Middleditch, Michael Booth, Richard Babor, Grant Beban, Rinki Murphy

**Affiliations:** 1 Maurice Wilkins Centre for Molecular Biodiscovery, University of Auckland, Auckland, New Zealand; 2 School of Biological Sciences, University of Auckland, Auckland, New Zealand; 3 Department of Medicine, University of Auckland, Auckland, New Zealand; 4 Department of Medicine, Department of Pharmacology and Pharmacy, The University of Hong Kong, Hong Kong, Special Administrative Region, China; 5 Department of Pharmacology, University of New South Wales, Sydney, New South Wales, Australia; 6 Department of Surgery, North Shore Hospital, Auckland, New Zealand; 7 Department of Surgery, Middlemore Hospital, Auckland, New Zealand; 8 Department of Surgery, Auckland City Hospital, Auckland, New Zealand; University of Nebraska Medical Center, United States of America

## Abstract

**Background:**

Bypass of foregut secreted factors promoting insulin resistance is hypothesized to be one of the mechanisms by which resolution of type 2 diabetes (T2D) follows roux-en-y gastric bypass (GBP) surgery.

**Aim:**

To identify insulin resistance-associated proteins and metabolites which decrease more after GBP than after sleeve gastrectomy (SG) prior to diabetes remission.

**Methods:**

Fasting plasma from 15 subjects with T2D undergoing GBP or SG was analyzed by proteomic and metabolomic methods 3 days before and 3 days after surgery. Subjects were matched for age, BMI, metformin therapy and glycemic control. Insulin resistance was calculated using homeostasis model assessment (HOMA-IR). For proteomics, samples were depleted of abundant plasma proteins, digested with trypsin and labeled with iTRAQ isobaric tags prior to liquid chromatography-tandem mass spectrometry analysis. Metabolomic analysis was performed using gas chromatography-mass spectrometry. The effect of the respective bariatric surgery on identified proteins and metabolites was evaluated using two-way analysis of variance and appropriate post-hoc tests.

**Results:**

HOMA-IR improved, albeit not significantly, in both groups after surgery. Proteomic analysis yielded seven proteins which decreased significantly after GBP only, including Fetuin-A and Retinol binding protein 4, both previously linked to insulin resistance. Significant decrease in Fetuin-A and Retinol binding protein 4 after GBP was confirmed using ELISA and immunoassay. Metabolomic analysis identified significant decrease of citrate, proline, histidine and decanoic acid specifically after GBP.

**Conclusion:**

Greater early decrease was seen for Fetuin-A, Retinol binding protein 4, and several metabolites after GBP compared to SG, preceding significant weight loss. This may contribute to enhanced T2D remission observed following foregut bypass procedures.

## Introduction

The success of bariatric surgery in obtaining remission of type 2 diabetes (T2D) varies with the type of surgery performed, with generally higher rates observed after gastric bypass (GBP) or biliopancreatic diversion, than with purely restrictive operations such as gastric banding [1], [2]. This is not explained by the difference in overall weight loss achieved by the different procedures, because weight loss achieved is not significantly associated with T2D outcome in individuals and there is no difference in weight loss between patients with remission of T2D and non-remission between or within bariatric procedures [3]. One of the mechanisms by which procedures involving intestinal rearrangement such as GBP and biliopancreatic diversion normalize glycaemia is through greater improvement in insulin resistance, seen to a lesser extent following non diversionary surgeries such as sleeve gastrectomy (SG) or gastric banding [4], [5].

To explain the rapid and sustained impact of GBP on improving glycaemia in patients with type 2 diabetes, two hypotheses have been proposed: The hindgut hypothesis suggests that the rapid transit of nutrients to the distal bowel improves glucose metabolism by stimulating secretion of the incretin hormone GLP-1 from the ileum and colon L-cells [6]. Indeed, prandial GLP-1 secretion acts to increase glucose-stimulated insulin secretion and improves post-prandial glycaemia, however this does not explain the rapid normalization of fasting glycaemia seen following GBP [7]. The foregut hypothesis proposes that nutrient bypass of the upper gut leads to reduction in secretion of an unidentified gut peptide which promotes insulin resistance [8], [9]. Identification of such a gut peptide could yield insights into the pathophysiology of obesity associated T2D and new therapeutic targets.

Proteomics refers to the identification and/or quantification of the proteins expressed in a tissue, and is used for both inter- and intra-individual comparisons of samples [10]. Early proteomic studies often relied on two-dimensional polyacrylamide gel electrophoresis [11] while newer proteomic approaches e.g. isobaric tagging of peptides separated by liquid chromatography and analyzed using tandem mass spectrometry have been developed to overcome limitations associated with gel electrophoresis and allow more rapid and accurate proteomic analysis of complex proteinaceous samples. New opportunities for discovery in biofluids have also opened up with the development of selective depletion strategies for highly abundant proteins which otherwise tend to dominate the analysis, obstructing the analysis of biomarkers and other key proteins that are often in low abundance [11]. Complementing proteomic techniques, metabolomic analysis offers a highly sensitive means to profile a large number of metabolites in various metabolic pathways [12] and provides useful information on activity of metabolic pathways e.g. lipid and carbohydrate metabolism [13], [14].

In this study we combined proteomic and metabolomic approaches to explore the foregut hypothesis. We aimed to identify proteins as well as metabolites which were decreased following GBP (foregut bypassed from nutrient flow), but not after SG (foregut intact with nutrient flow), with special focus on molecules already linked to insulin resistance. The post-operative time point (3 days post-operatively) was carefully chosen in order to place focus on cause rather than effect. Three days after surgery the greater expected T2D remission following GBP than SG was still negligible, hence any significant differences found in the plasma proteomic and metabolomic profiles were unlikely resulting from T2D remission. The effect of bypassing the foregut on secretion of molecules should however be evident shortly after GBP. By performing within-patient comparisons of fasting plasma protein and metabolite profiles before and soon after either GBP or SG when post–surgical caloric intake and physical activity was equivalent and very little weight loss had occurred, we also avoided confounding effects of weight loss and differences in caloric intake.

## Materials and Methods

**Figure 1:**
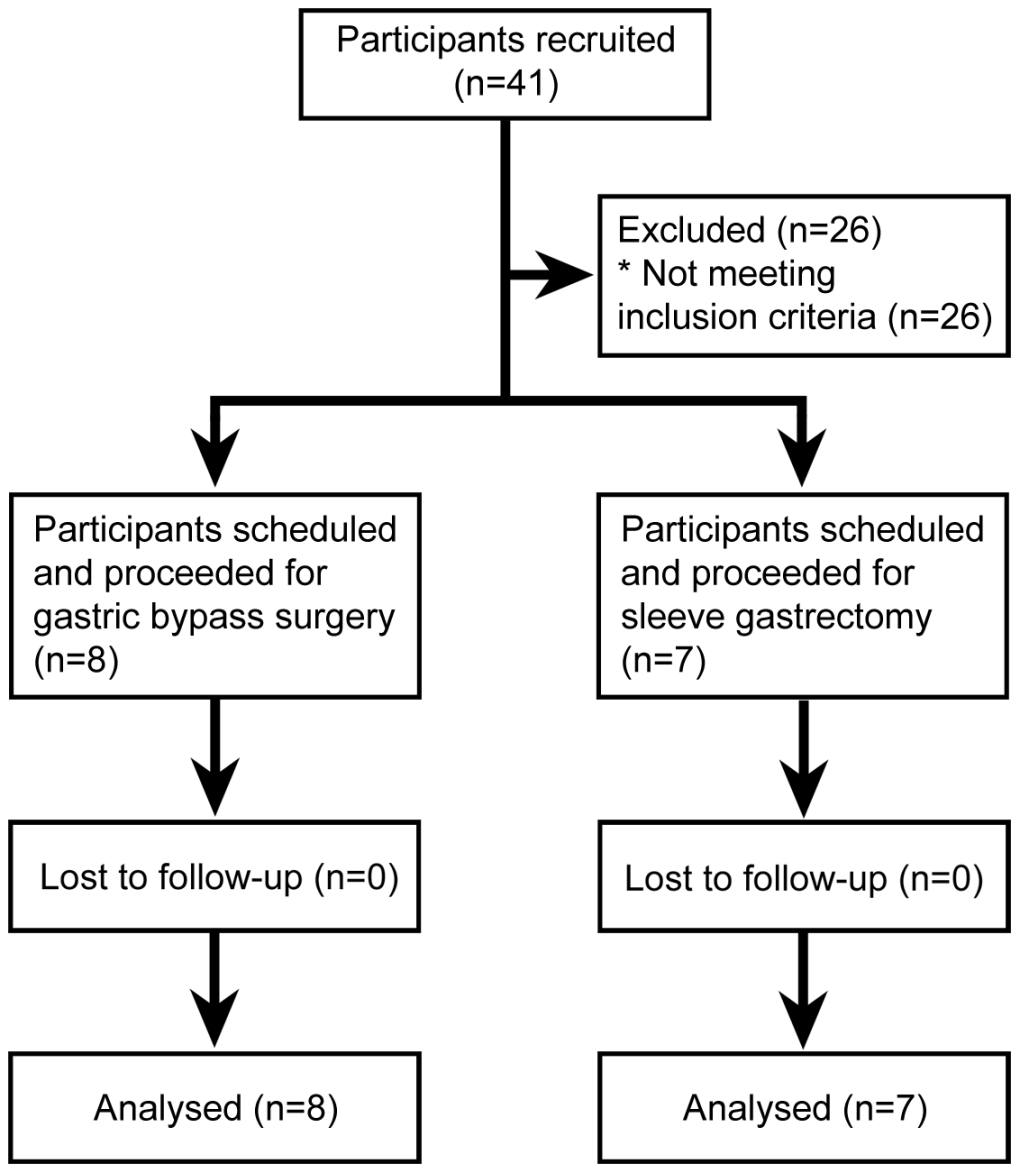
2010 CONSORT flow diagram for the study.

A non-randomised, matched, prospective controlled intervention trial that compared the acute effect of GBP to SG, compared with matched caloric intake, on glycemia among obese patients with type 2 diabetes has been previously described with its inclusion and exclusion criteria [15]. Briefly, from 1 August 2010 to 31 March 2012, 21 obese patients with type 2 diabetes treated with oral glucose lowering therapies (but not incretin hormone therapies) were recruited by contacting patients on the bariatric surgery waiting lists, 10 who were scheduled for SG and 11 who were scheduled for GBP. A total of 7 patients who had SG and 8 who had GBP, all treated with metformin monotherapy at baseline, were eligible for inclusion in this pilot proteomic and metabolomic study. Baseline and follow up assessments were conducted in the patients' homes at 3 pre- and post-operative days. The protocol, allocation and supporting TREND checklist for this study are available as supporting information (Checklist S1 and Protocol S1), and Figure 1 for the allocation flow chart. All patients were prescribed a hypocaloric diet with three servings of Optifast (152 Calories) per day during the three weeks prior to surgery and were instructed to fast from midnight the night before surgery. Patients received general anaesthesia with atracurium and propofol or equivalent agents for both laparoscopic surgeries. The GBP consisted of a 100 cm antecolic Roux-limb with hand-sewn pouch-jejunostomy, a 60 cm bilio-pancreatic limb and a hand sewn small bowel anastomosis. The SG was performed by a longitudinal resection of the stomach against a 32 French bougie from just lateral to the angle to His to 2 cm proximal to the pylorus.

Post-operative IV fluids with Plasmalyte were administered until oral fluid intake was 1 litre per day (approximately 48 hours). Routine medications given for the first 48 hours included analgesia as required (paracetamol, tramadol and fentanyl), anti-emetics (ondansetron, cyclizine and metoclopramide), and subcutaneous heparin as prophylaxis for venous thrombo-embolism. Metformin therapy was stopped from the day of surgery until the end of the study period. Standard post-operative oral intake instructions were as follows: Day 1, sips of water; Day 2-4, free oral fluids with clear soups, low fat smoothies or Optifast.

Approximately 3 days before surgery, and again 3 days after surgery, in both cases following fasting for at least 10 hours, blood samples were collected into EDTA tubes and centrifuged following routine procedures to yield plasma samples which were stored at −80°C until analysis.

### Ethics statement

This prospective study was approved by the Northern X Regional Ethics Committee, New Zealand and was registered with the Australian New Zealand Clinical Trials Registry (ACTRN12609000679280). Informed, written consent was obtained from all patients.

### Metabolic parameters

Serum insulin and glucose were quantified using the Human Metabolic Hormone Panel (Milliplex, Millipore) and Hitachi 902 auto-analyzer (Hitachi High Technologies Corporation), respectively. HOMA-IR was calculated as (fasting insulin in µU/ml x fasting glucose in mmol/L)/22.5.

### Proteomic procedures

Plasma samples were subjected to immunodepletion of 20 highly abundant serum proteins (albumin, immunoglobulins (IgG, IgM, IgD), transferrin, fibrinogen, α-1-antitrypsin, α-2-antitrypsin, complements C3, C4, C1q, haptoglobin, apolipoproteins (A1, A2, B), acid-1-glycoprotein, ceruloplasmin, prealbumin, plasminogen) using a ProteoPrep 20 plasma immunodepletion centrifugal device (Sigma Aldrich, St Louis, MO, USA,) according to the manufacturer's instructions. Depleted samples were stored at −80°C until further processing. Sample volumes containing 40 µg total protein were reduced at 55°C for 15 minutes using 10 mM dithiothreitol, alkylated with 50 mM iodoacetamide (Sigma) at room temperature for 45 minutes in the dark and then digested at 40°C for 30 minutes with 2 µg of sequencing grade trypsin (Promega, Madison, WI, USA) in a chilled microwave (CEM Corporation, Matthews, NC, USA). Digests were cleaned up on 10 mg Oasis HLB SPE cartridges (Waters, Milford, MA, USA), eluted with 300 µl of 60% acetonitrile, dried in a vacuum centrifuge (Thermo Savant, Holbrook, NY, USA) and reconstituted with 20 µl of iTRAQ dissolution buffer (0.5 M triethylammonium bicarbonate at pH 8.5; Applied Biosystems, Foster City, CA, USA). Two samples from each subject (pre- and post- surgery) were derivatised with one of eight iTRAQ labels and combined in groups of eight. Pooled samples were separated by multidimensional liquid chromatography using a strong cation exchange column, followed by a reversed phase column (150 mm×300 um Zorbax SB 300A C-18 column, Agilent Technologies, Santa Clara, CA, USA). The column effluent was analyzed on a QSTAR-XL hybrid mass spectrometer (Applied Biosystems). A TOF-MS scan was made from m/z 300-1600 and the three most intense multiply-charged precursors isolated for fragmentation, with MS/MS spectra recorded from m/z 75-1600.

The patient samples were first analyzed in groups of eight as described above across four liquid chromatography-tandem mass spectrometry (LC-MS/MS) runs; an additional LC-MS/MS run containing selected samples from all four individual runs was then conducted to enable comparisons between runs.

The LC-MS/MS outputs were used to search the human IPI database (Version 3.87) using ProteinPilot software version 4.0 (Applied Biosystems) and protein matches with scores above the 1% false discovery rate threshold for each run were accepted for quantitative analysis. In cases where the software identified an unnamed or putative protein from the database, the amino acid sequence of the protein was used to conduct a BLAST search (available online at http://blast.ncbi.nlm.nih.gov/Blast.cgi) to gain a more meaningful identifier.

The ProteinPilot peptide summaries were used to manually calculate the abundance of each protein in each sample relative to a pre-GBP sample included in the same run as previously described [16]. By comparing in the additional run all pre-GBP samples that were used as reference samples within the previous runs, results obtained from separate LC-MS/MS runs could be scaled and effectively used to compare all samples across all runs. Finally, average ratios calculated in log space for each group were converted to linear space for presentation. Confidence intervals were calculated by converting to linear space the average ratios (ln) ± SEM (ln). Data files containing the complete proteomic output are available from the authors upon request.

Proteins of interest were validated using immunoassay or ELISA. Retinol Binding Protein 4 (RBP4) was measured as per manufacturer's protocol using the Human RBP4 Immunoassay Kit (AIS antibody and Immunoassay Services, Hong Kong). Fetuin-A were measured by a sandwich ELISA (BioVendor Laboratory Medicine, Brno, Czech Republic).

### Metabolomics procedures

For each sample, a 300 µL aliquot of plasma was spiked with 20 µL of internal standard (10 mM d4-alanine; 2,3,3,3-d4-dl-alanine, 98 atom % D; Sigma-Aldrich), then lyophilised overnight before being extracted first with 500 µL of 50% methanol, then with 500 µL of 80% methanol. Extracted supernatants were pooled and freeze-dried. Dried samples were chemically derivatised by methyl chloroformate and analyzed by Gas Chromatography-Mass spectrometry (Agilent 7890A coupled with a 5975 inert Mass Spectrometry Detector). Chromatograms were processed using an in-house metabolite library and a custom-made R-software package, with manual checking and correction of information (GC-MS peak intensities). Metabolomic data for each sample were normalized using the internal standard prior to calculation of relative abundances of the separate metabolites in the four different groups.

### Statistical analysis

All statistical calculations were performed using GraphPad Prism version 6.00 (GraphPad Software, La Jolla California USA).

Statistical significance of the effect of bariatric surgery on fasting glucose, fasting insulin and HOMA-IR was assessed using repeated measures, mixed model two-way ANOVA, pairing each post-operative sample with the pre-operative sample from the same patient.

Statistical analysis of the proteomic data was done after conversion of all ratios to their natural logs, as customary for iTRAQ data. The separate effect of surgery (post- versus pre-) as well as surgery group (GBP versus SG) were calculated for all identified proteins using repeated measures, mixed model two-way ANOVA paired as above. All proteins showing significant effect of bariatric surgery (p<0.05), were further evaluated using Sidak's multiple comparisons post-hoc test to test the specific effect of GBP and SG on protein levels.

The results of the immunoassay and ELISA validations were tested using two-tailed one sample t tests.

For metabolomic data, the separate effects of bariatric surgery as well as surgery type were calculated for all identified metabolites using repeated measures, mixed model two-way ANOVA. Metabolites showing significant effect of bariatric surgery (p<0.05) were further evaluated using Sidak's multiple comparisons post-hoc test to test the specific effect of GBP and SG on metabolite levels.

Correlation between selected proteins/metabolites and HOMA-IR was evaluated using Spearman correlations.

## Results

### Clinical characteristics

Fifteen obese subjects participating in a prospective bariatric surgery study [15] (8 scheduled for GBP, 7 for SG) were selected for this study based on similarity of baseline characteristics (Table 1). All patients were treated with metformin for T2D prior to surgery only. Despite cessation of metformin therapy post-operatively, mean fasting glucose and insulin levels, as well as insulin resistance (HOMA-IR) improved after both GBP and SG, although the differences did not reach statistical significance (Table 1).

**Table 1:** Subject characteristics and effect of surgeries on fasting glucose, fasting insulin and HOMA-IR.

	GBP (n = 8)		SG (n = 7)	
**Sex**	Females: 8		Females: 6	
	Males: 0		Males: 1	
**Age** (yrs)	41.0±3.1		46.8±2.9	
**Ethnicity**	5 NZE		6 NZE	
	2 PI		1 PI	
	1 Asian			
**Duration diabetes** (yrs)	3.7±0.7		3.1±0.6	
**BMI** (kg/m^2^)	42.1±4.0		42.3±5.9	
**HbA1c** (%)	7.2±0.2		7.8±0.7	
(mmol/mol)	54.8±2.5		61.9±7.8	
**Duration of surgery** (hours)	2.60±0.08		2.14±0.01	
	Baseline	Day 3 post surgery	Baseline	Day 3 post surgery
**Fasting glucose** (mmol/L)	6.9±0.7	5.9±0.5	7.0±0.7	6.1±0.6
p (pre Vs post bariatric surgery): 0.06				
p (SG Vs GBP): 0.82				
**Fasting insulin** (µIU/mL)	17.5±6.9	10.8±2.7	20.6±5.6	16.9±6.0
p (pre Vs post bariatric surgery): 0.15				
p (SG Vs GBP): 0.53				
**Log HOMA-IR**	1.07±0.45	0.75±0.32	1.64±0.25	1.26±0.31
p (pre Vs post bariatric surgery): 0.18				
p (SG Vs GBP): 0.23				

Data are shown as mean ± S.E.M.; p values are derived from Mixed Model Repeated Measures Two-Way ANOVA. Abbreviations: NZE, NZ European; PI, Pacific Islander; HOMA-IR, insulin resistance calculated by homeostatic model assessment.

### Proteomic analysis

**Figure 2:**
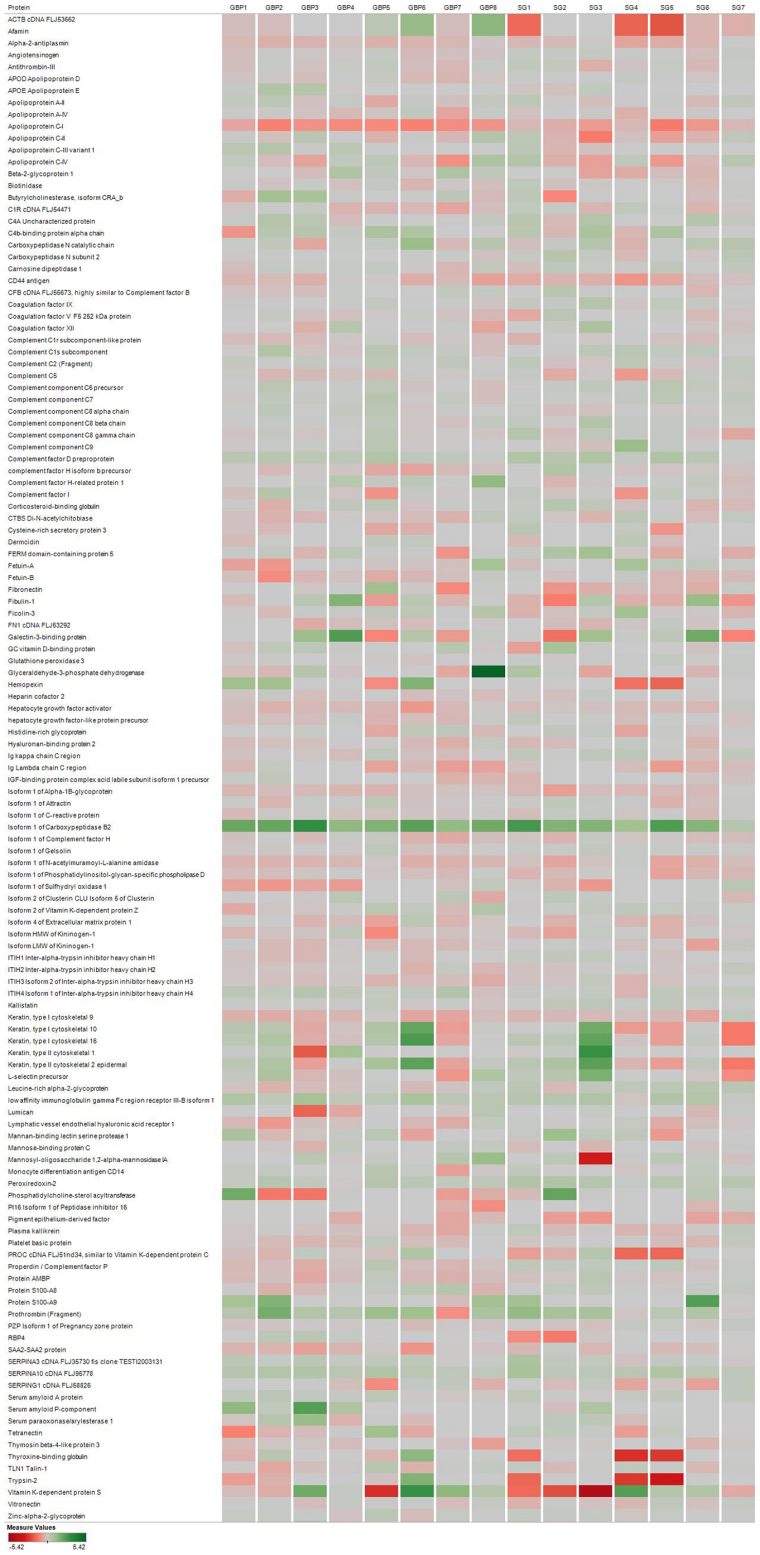
Heat map showing patient specific changes of all detected proteins. Each protein is represented on an individual row with data from each patient stacked in columns (GBP1 to GBP8 for GBP, SG1-SG7 for SG). Intensity of color corresponds to the degree of change from red (decrease) to green (increase) after surgery.

**Figure 3:**
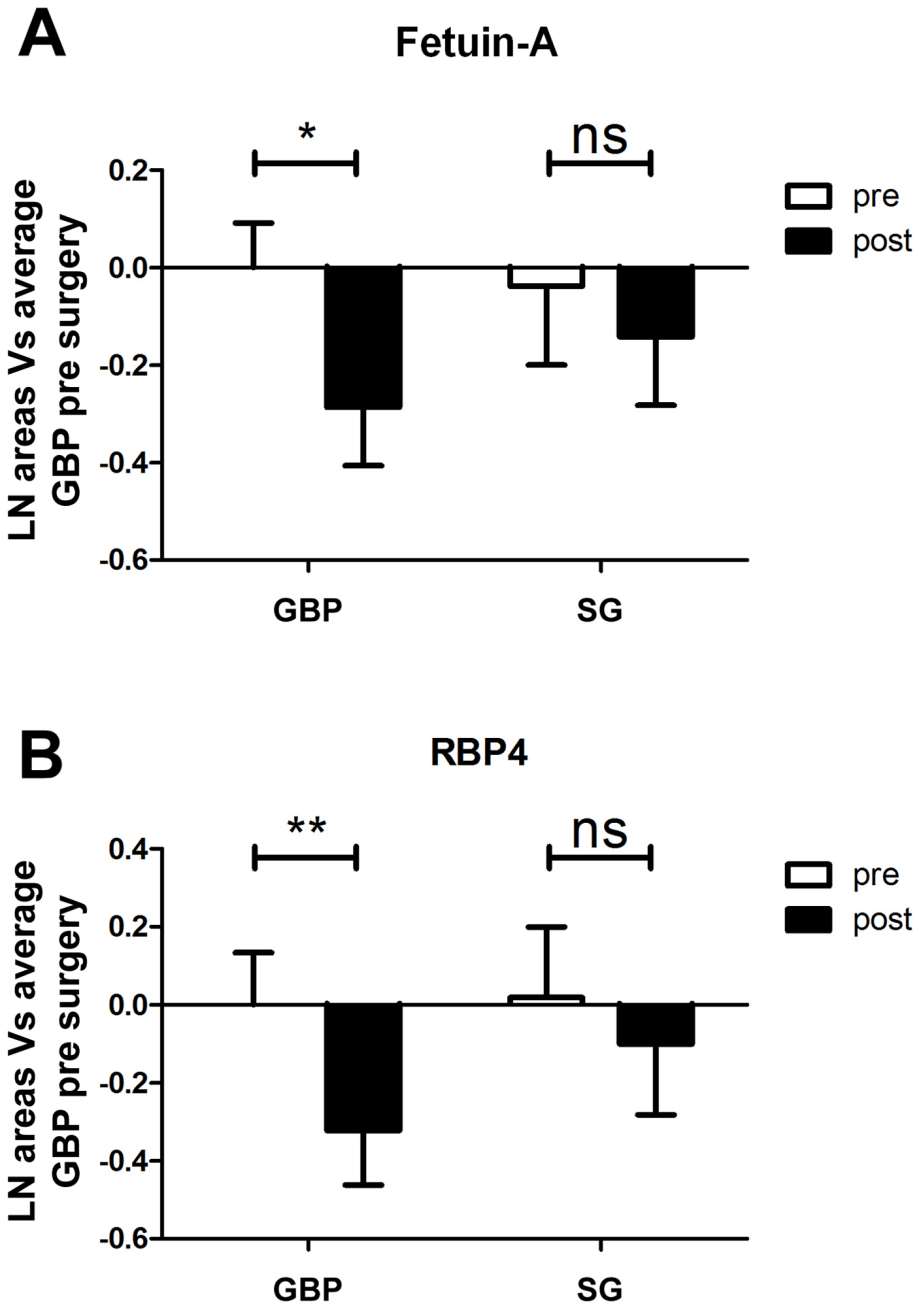
Proteomic results for Fetuin-A and RBP4. Relative abundance pre and post GBP and SG for Fetuin-A (A), and RBP4 (B) as per iTRAQ proteomic study, showing significant decrease after GBP but not after SG for both proteins. Abbreviations: *, p<0.05; **, p<0.01.

**Figure 4:**
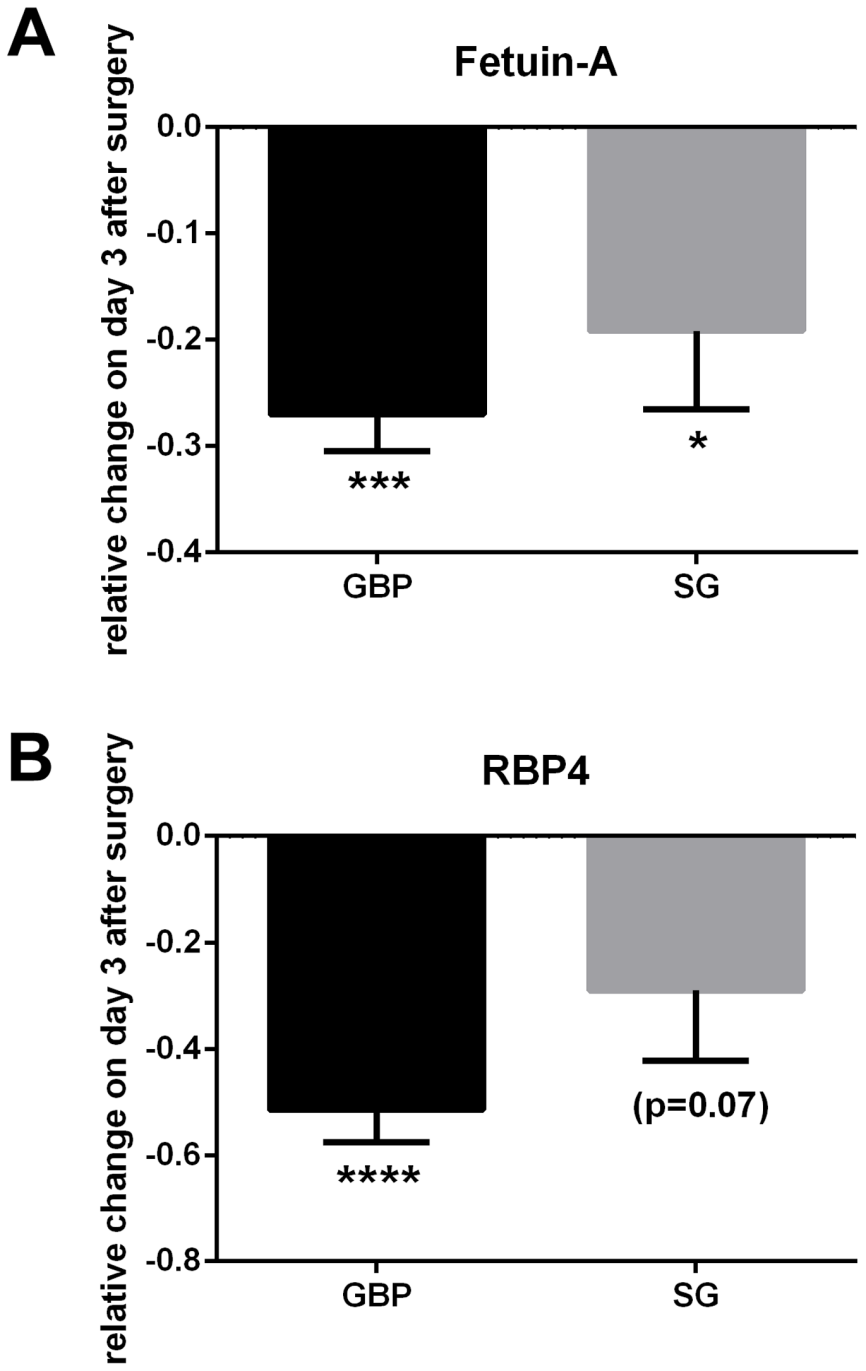
Validation of proteomic results for Fetuin-A and RBP4. Panel A: Fetuin-A levels were found by ELISA to decrease to some extent after both surgery types, however a more dramatic decrease was found after GBP. Panel B: significantly lower levels of RBP4 were detected by immunoassay after GBP only. Abbreviations: *, p<0.05; ***, p<0.001; ****, p<0.0001.

Of a total of 85 proteins identified with sufficient iTRAQ label and in three or more individuals per surgery group (Fig. 2), two-way ANOVA analysis showed significant effect of bariatric surgery for 32 proteins when time (before vs. after surgery) was plotted against surgery type (GBP vs SG). Of these, 23 proteins were significantly different also in post hoc tests, where the effects of the two surgery types were analyzed separately (Table 2). Summary statistics for all 85 identified proteins are available in Table S1. Specifically, six proteins were significantly lower and four significantly higher after both surgery types, four proteins increased and one decreased after SG only while only one protein increased after GBP. Seven proteins were significantly lower after GBP only and hence matched the main aim of the study. Of these, two (RBP4 and Fetuin-A) were previously reported in the context of insulin resistance. The significant decrease in RBP4 (decreased to 72% after GBP, p<0.01) and Fetuin-A (decreased to 75% after GBP, p<0.05) as demonstrated by the iTRAQ proteomic study is detailed in Figure 3. Validation analysis by immunoassay and ELISA confirmed significant decrease of RBP4 (−51.5±6.1%, p<0.0001) and Fetuin-A (−27.0±3.5%, p = 0.0001) on day 3 after GBP (Fig. 4); consistent with the iTRAQ results. As shown in Table 3, the measured levels of both RBP4 and Fetuin-A showed significant correlation with HOMA-IR.

**Table 2:** Proteins displaying post-operative changes in relative abundance (3 days after GBP and/or SG compared to 3 days prior to surgery) according to the proteomic study.

Protein name (Accession No)	pre GBP/pre GBP	post GBP/pre GBP	pre SG/pre GBP	post SG/pre GBP
***Significant change after GBP only***				
Isoform 2 of Inter-alpha-trypsin inhibitor heavy chain H3 (IPI00876950.1)	1.00	**1.33** **	1.15	**1.29** (n.s.)
	(0.88–1.14)	(1.16–1.54)	(1.00–1.32)	(1.09–1.53)
RBP4 (retinol-binding protein 4) (IPI00844536.2)	1.00	**0.72** **	1.02	**0.90** (n.s.)
	(0.87 –1.14)	(0.63–0.83)	(0.85–1.22)	(0.75–1.09)
Alpha-2-HS-glycoprotein ( = Fetuin-A) (IPI00953689.1)	1.00	**0.75** *	0.96	**0.87** (n.s.)
	(0.91–1.10)	(0.67–0.85)	(0.82–1.13)	(0.75–1.00)
Coagulation factor X (IPI00019576.1)	1.00	**0.83** *	1.07	**0.98** (n.s.)
	(0.87–1.14)	(0.71–0.96)	(0.87–1.32)	(0.78–1.23)
Coagulation factor XII (IPI00019581.2)	1.00	**0.88** *	0.96	**0.87** (n.s.)
	(0.87–1.15)	(0.75–1.03)	(0.84–1.10)	(0.76–1.00)
Heparin cofactor 2 (IPI00879573.1)	1.00	**0.74** ***	1.18	**1.03** (n.s.)
	(0.90–1.11)	(0.67–0.82)	(1.04–1.35)	(0.89–1.19)
Inter-alpha-trypsin inhibitor heavy chain 2 (IPI00645038.1)	1.00	**0.81** **	1.05	**0.97**(n.s.)
	(0.94–1.06)	(0.74–0.89)	(1.01–1.08)	(0.91–1.04)
Tetranectin (IPI00009028.2)	1.00	**0.80** *	0.90	**0.82** (n.s.)
	(0.91–1.10)	(0.75–0.85)	(0.80–1.02)	(0.76–0.89)
***Significant change after SG only***				
Complement C5 (IPI00032291.2)	1.00	**1.07** (n.s.)	1.00	**1.21** *
	(0.92–1.08)	(0.99–1.15)	(0.91–1.09)	(1.11–1.31)
Complement component C8 alpha chain (IPI00011252.1)	1.00	**1.06** (n.s.)	1.03	**1.24** *
	(0.91–1.10)	(0.97–1.16)	(0.94–1.12)	(1.11–1.39)
CFB cDNA FLJ55673, (highly similar to Complement factor B) (IPI00019591.2)	1.00	**1.04** (n.s.)	0.98	**1.17** *
	(0.93–1.08)	(0.97–1.12)	(0.91–1.07)	(1.07–1.29)
Isoform 1 of Inter-alpha-trypsin inhibitor heavy chain H4 (IPI00896419.3)	1.00	**1.04** (n.s.)	0.97	**1.14** **
	(0.93–1.07)	(0.98–1.10)	(0.90–1.04)	(1.08–1.20)
N-acetylmuramoyl-L-alanine amidase (IPI00163207.1)	1.00	**0.89** (n.s.)	1.09	**0.85** **
	(0.88–1.14)	(0.77–1.04)	(0.96–1.23)	(0.73–0.98)
***Significant change after both surgery types***				
Isoform 1 of C-reactive protein (IPI00022389.1)	1.00	**7.79** ****	0.78	**4.32** ****
	(0.82–1.22)	(6.07–9.99)	(0.61–1.01)	(3.47–5.37)
SERPINA3 (highly similar to alpha-1-antichymotrypsin) (IPI01025667.1)	1.00	**1.61** ****	0.92	**1.46** ****
	(0.76–1.31)	(1.24–2.10)	(0.68–1.24)	(1.04–2.06)
Complement component C9 (IPI00022395.1)	1.00	**1.43** ***	0.90	**1.42** ***
	(0.92–1.09)	(1.34–1.51)	(0.80–1.00)	(1.36–1.49)
Leucine-rich alpha-2-glycoprotein (IPI00022417.4)	1.00	**1.70** ***	0.80	**1.23** **
	(0.91–1.10)	(1.51–1.91)	(0.70–0.91)	(1.12–1.34)
Apolipoprotein A-IV (IPI00304273.2)	1.00	**0.40** ****	0.85	**0.49** ***
	(0.86–1.16)	(0.34–0.46)	(0.78–0.93)	(0.42–0.55)
Insulin-like growth factor-binding protein complex acid labile subunit (IPI00925635.1)	1.00	**0.81** **	0.81	**0.63** **
	(0.95–1.05)	(0.75–0.86)	(0.76–0.87)	(0.57–0.69)
Beta-Ala-His dipeptidase (Carnosine dipeptidase 1) (IPI00064667.5)	1.00	**0.75** **	1.11	**0.74** ***
	(0.91–1.10)	(0.67–0.84)	(0.98–1.25)	(0.65–0.84)
Kallistatin (IPI00328609.3)	1.00	**0.70** ***	1.11	**0.90** *
	(0.95–1.05)	(0.66–0.75)	(1.01–1.22)	(0.85–0.95)
Gelsolin (IPI00026314.1)	1.00	**0.74** ***	0.89	**0.69** **
	(0.94–1.07)	(0.68–0.80)	(0.81–0.98)	(0.68–0.70)
Afamin (IPI00019943.1)	1.00	**0.76** ***	1.05	**0.80** **
	(0.94–1.07)	(0.70–0.82)	(0.97–1.14)	(0.74–0.88)

All listed proteins displayed significant (p<0.05) effect of surgery (SG and GBP) as tested by two-way analysis of variance. IPI numbers in brackets refers to accession numbers in the human IPI database; asterisks following ratios denote significant effect of the indicated surgery type according to post-hoc test;

*, p<0.05;

**, p<0.01;

***, p<0.001;

****, p<0.0001; n.s., not significant. Values in brackets refer to confidence intervals.

**Table 3:** Assessment of correlation between selected proteins and metabolites (showing greater decrease after GBP than SG) with insulin resistance (estimated by HOMA-IR).

	Fetuin-A	RBP4	Proline	Citrate	Histidine	Decanoic acid
HOMA-IR	0.36	0.48	0.10	0.06	–0.04	–0.07
p	(<0.05)*	(0.006)**	(0.61)	(0.74)	(0.85)	(0.72)

The values shown are Spearman rank correlation coefficients with two-tailed p values for significance of correlation in brackets. Asterisks highlights significant correlations (*, p<0.05; **, p<0.01).

### Metabolomic analysis

**Figure 5:**
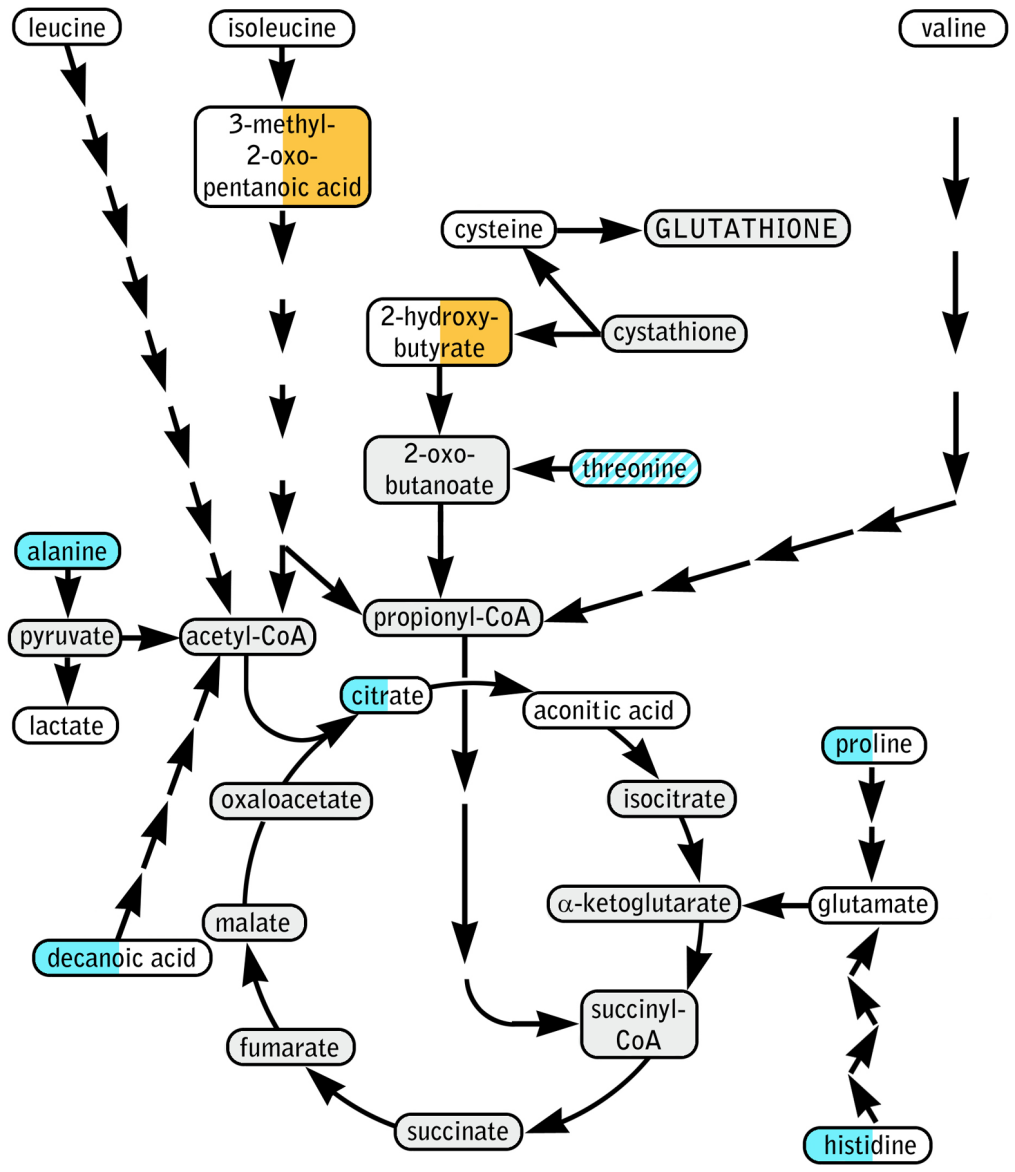
Schematic of implicated metabolic pathways based on metabolomics results (selected metabolites only). Metabolites are inscribed in color coded ellipses with the effect of GBP in left half and effect of SG in right half. Color code: blue, significantly lower; orange, significantly higher; white, no significant effect of bariatric surgery; diagonal stripes, significant effect of surgery but not significant in post-hoc test. Grey highlights an undetected metabolite.

A total of 45 metabolites were detected, of which 19 were fatty acids and 20 were amino acids or amino acid derivatives. Eight metabolites showed significant (p<0.05) impact of bariatric surgery in the two-way ANOVA; post hoc analysis detected significant effect of one or both specific surgery types for seven of these (Table 4). Figure 5 shows significantly affected and other relevant metabolites in their implicated pathways. Summary statistics for all 45 detected metabolites are available in Table S2.

**Table 4:** Metabolites showing significant effect (p<0.05) of bariatric surgery in a Repeated Measures Mixed Model Two-Way ANOVA.

Metabolite	Pre-GBP	Post-GBP	Pre-SG	Post-SG	Effect of surgery (p)
	Mean ± SEM	Mean ± SEM	Mean ± SEM	Mean ± SEM	
Alanine	1.00±0.09	**0.52±0.04** *	1.27±0.19	**0.68±0.06** **	0.0001
Proline	1.00±0.12	**0.56±0.09** *	1.00±0.18	0.71±0.11	0.0046
Histidine	1.00±0.12	**0.72±0.07** *	0.78±0.10	0.68±0.07	0.0073
Threonine	1.00±0.16	0.84±0.10	0.80±0.14	0.78±0.08	0.0452
Citric acid	1.00±0.10	**0.76±0.07** *	1.00±0.07	0.88±0.07	0.0095
Decanoic acid	1.00±0.12	**0.67±0.08** *	0.86±0.07	0.77±0.07	0.0232
2-Hydroxybutyric acid	1.00±0.14	1.16±0.17	0.97±0.12	**2.01±0.39** *	0.0359
3-Methyl-2-oxopentanoic acid	1.00±0.11	0.93±0.14	0.81±0.12	**1.57±0.24** **	0.0428

Values given are scaled to mean pre-GBP to enable comparison between all groups. Asterisks denote significant difference compared to baseline for the specific surgery type (Sidak's multiple comparisons test);

*, p<0.05;

**, p<0.01.

## Discussion

We hypothesized that key species promoting insulin resistance (proteins or their downstream metabolic targets) would be decreased following GBP, where the foregut is bypassed from nutrient flow, but not after SG, where the foregut remains intact to nutrient flow. To identify such causal metabolic factors, we chose to study subjects shortly after both types of surgery, specifically before significant weight loss had occurred and before differences in remission of diabetes between the two types of surgery were evident.

Amongst other findings, our proteomic analysis showed a significant decrease in two proteins involved in insulin resistance, RBP4 and Fetuin-A, [17], [18], three days after GBP but not SG. Notably, although insulin resistance had not improved significantly three days after bariatric surgery, the statistically significant correlations between the levels of RBP4 and Fetuin-A with HOMA-IR support a direct relationship between lower levels of these proteins and improved insulin resistance in our dataset.

RBP4 is a vitamin A (retinol) transport protein secreted by hepatocytes and adipocytes into the bloodstream. This protein carries retinol to peripheral tissues where it is converted into ligands for nuclear hormone receptors and regulates gene transcription. In recent years, RBP4 has been proposed to be a cardiometabolic risk factor associated with T2D, obesity and hyperlipidaemia [19] and it appears to induce insulin resistance in skeletal muscle, liver and adipose tissue in vitro [20]. The regulation of RBP-4 is unclear, however RBPR2 was recently identified as a high affinity RBP4 receptor expressed primarily in liver and small intestine and induced in adipocytes of obese mice [21]. Studies show that RBP4 is decreased with hypocaloric diet [22] as well as weight loss achieved by lifestyle [23] or weight loss achieved by SG bariatric surgery [12], [24].

Fetuin-A is produced in the liver and has a role in the inhibition of insulin-receptor tyrosine kinase, which attenuates insulin signalling and triggers insulin resistance [25], [26] and also down-regulates adiponectin, a known insulin sensitizer secreted by adipose tissue [27]. Fetuin-A has recently been shown to be an endogenous ligand for Toll-like receptor 4 (TLR4) through which it has a critical role in stimulating adipose tissue inflammation resulting in insulin resistance [28]. The regulation of Fetuin-A is thought to be through pro-inflammatory cytokines [29], [30]. Studies suggest that those with high Fetuin-A levels have increased risk of myocardial infarction [31], stroke [32], and incident diabetes [17]. Fetuin-A levels have also been reported to decrease after weight loss achieved by lifestyle [33] and after GBP [34].

The greater decrease of both Fetuin-A and RBP4 seen after GBP than after SG is consistent with an impact of foregut exclusion on reducing these proteins. In the case of RBP4, this may be because diversion of nutrient absorption past the duodenum resulted in reduced foregut absorption of retinol and expression of RBRP2 [21]. Alternatively, altered gut microbiota, which has been reported to occur after both types of surgery [35], [36], [37], may influence the levels of these plasma proteins. For example, distinct gut microbiota functional changes after GBP may produce less circulating endotoxin levels and thereby incite lower levels of Fetuin-A [37]. Further studies are required to document the functional evolution of gut microbiota after foregut excluding GBP compared to restrictive types of bariatric surgery such as SG in order to test these hypotheses.

Our metabolomic analysis highlighted several metabolites which responded differently to the two types of bariatric surgery. Specifically after GBP, we detected significantly lower levels of the glucogenic amino acids histidine and proline, as well as of citrate and decanoic acid. This response is consistent with improved metabolic status based on several points. Enhanced post-prandial disposal of amino acids and more complete beta-oxidation of fatty acids has been reported following GBP compared with caloric restriction [38]. Elevated free fatty acids are well known to be associated with insulin resistance [39] and decreased decanoic acid after GBP may be a result of a greater uptake of long chain fatty acid into adipocytes [40]. Elevated histidine [41], [42] and citrate [43], [44], [45], [46] levels have been linked with insulin resistance and T2D. Although we did not find any correlation between these four metabolites and insulin sensitivity at this early stage, it is possible they may be predictive of future improvements.

Conversely, after SG we detected marked elevation of the branched-chain amino acid catabolite 3-methyl-2-oxo-pentanoic acid, accompanied by a non-significant tendency towards increased levels of all branched-chain amino acids. The latter is a well-known phenomenon in insulin resistance [47]. Also substantially elevated following SG was 2-hydroxybutyrate, which has been proposed as a plasma biomarker for early stages of diabetes [48] and insulin resistance [49].

Together, the differential early changes in the above metabolites following GBP and SG suggest a more favourable metabolic profile associated with greater insulin sensitivity early after GBP compared with SG. Since both surgery groups were of similar obesity and diabetes status as well as similar in terms of reduced caloric intake at the post-operative time-point, these differences are likely directly linked to the types of bariatric surgery performed.

This study has two major limitations. Firstly, it is limited by small sample size. The subjects were however well matched for baseline clinical characteristics before two relatively similar laparoscopic bariatric surgical procedures with well-matched post-operative protocols for analgesia, fluid replacement and caloric intake. Secondly, the immunodepletion method used in the proteomic analysis improved detection of moderately abundant proteins but had limited ability to prime the samples for detection of proteins of very low abundance in plasma. Depletion techniques targeting a larger number of proteins [50] would allow a more comprehensive analysis in the search for additional novel biomarkers of insulin resistance. Nonetheless, our proteomic study identified significant decreases in RBP4 and Fetuin-A concentrations after GBP but not after SG, which was confirmed using other analytical methods. The metabolomic analysis was also limited to a relatively moderate number of metabolites and future investigations will benefit from the use of more powerful equipment.

Although we were unable to obtain a clear connection between our proteomic and metabolomics findings, by using these two complementary analytical approaches, we managed to identify a number of potentially important changes in key metabolic pathways as well as proteins with known or putative roles in the development of insulin resistance.

In summary, our findings suggest greater lowering of multiple proteins including RBP4, Fetuin-A as well as several metabolites associated with insulin resistance following GBP compared with SG prior to diabetes remission.

## Supporting Information

Table S1 **Summary statistics for all identified proteins.** Relative abundance, confidence intervals and outcome of statistical analysis are shown for the 85 proteins identified with sufficient confidence and iTRAQ label in at least 3 within-patient comparisons as well as the final LC-MS/MS run combining samples from all previous runs. Entries in bold showed significant effect of surgery according to the Two-way ANOVA; *, p<0.05; **, p<0.01; ***, p<0.001, ****, p<0.0001; t, trending (p<0.10); n.s., p>0.10.
(DOCX)

Table S2 **Summary statistics for all identified metabolites.** Abundance (relative to averaged pre-operative GBP samples), S.E.M. and outcome of statistical analysis (Two-Way ANOVA followed by Sidak's post-test) are shown for all 45 metabolites identified. Abbreviations: *, p<0.05; **, p<0.01; ***, p<0.001, ****, p<0.0001; n.s., p>0.05.
(DOCX)

Checklist S1 **TREND Checklist used in this study.**
(DOCX)

Protocol S1 **Proteomic sub-study protocol detailing patient recruitment, exclusion criteria and study protocol.**
(DOCX)
