# Supplementary material for: Lower Fetuin-A, Retinol Binding Protein 4 and Several Metabolites after Gastric Bypass Compared to Sleeve Gastrectomy in Patients with Type 2 Diabetes
Source: PLoS One. 2014 May 6;9(5):e96489. doi: 10.1371/journal.pone.0096489 (PMC4011803; doi:10.1371/journal.pone.0096489)
Supplement: Table S1 — Summary statistics for all identified proteins. Relative abundance, confidence intervals and outcome of statistical analysis are shown for the 85 proteins identified with sufficient confidence and iTRAQ label in at least 3 within-patient comparisons as well as the final LC-MS/MS run combining samples from all previous runs. Entries in bold showed significant effect of surgery according to the Two-way ANOVA; *, p<0.05; **, p<0.01; ***, p<0.001, ****, p<0.0001; t, trending (p<0.10); n.s., p>0.10. (DOCX) [file pone.0096489.s001.docx]

**Table S1**

**Summary statistics for all identified proteins.** Relative abundance, confidence intervals and outcome of statistical analysis are shown for all 85 proteins identified with sufficient confidence and iTRAQ label in at least 3 within-patient comparisons as well as the final LC-MS/MS run combining samples from all previous runs. Entries in bold showed significant effect of surgery according to the Two-way ANOVA; *, p<0.05; **, p<0.01; ***, p<0.001, ****, p<0.0001; t, trending (p<0.10); n.s., p>0.10.

|  |  |  | **Two-ANOVA** (*p*) | | | | **Post-hoc test (post Vs pre)** | | **GB pre** | | | **GB post** | | | **n** | **SG pre** | | | **SG post** | | | **n** |
| --- | --- | --- | --- | --- | --- | --- | --- | --- | --- | --- | --- | --- | --- | --- | --- | --- | --- | --- | --- | --- | --- | --- |
|  | **Accessions** | **Protein names** | Interaction | surgery (type) | surgery (post Vs pre) | Subjects (matching) | GB (*p*) | SG (*p*) | **Average** | min | max | **Average** | min | max | GB post/ GB pre | **Average** | min | max | **Average** | min | max | SG post/ SG pre |
| **Acute phase response** | | | | | | | | | | | | | | | | | | | | | | |
|  | IPI00022389.1 | **CRP Isoform 1 of C-reactive protein** | n.s. | n.s. | **** | t | **** | **** | **1.00** | 0.82 | 1.22 | **7.79** | 6.07 | 9.99 | **8** | **0.78** | 0.61 | 1.01 | **4.32** | 3.47 | 5.37 | **7** |
|  | IPI01025667.1 IPI01025667.1  IPI00847635.1 | **SERPINA3 cDNA FLJ35730 fis, clone TESTI2003131, highly similar to ALPHA-1-ANTICHYMOTRYPSIN** | n.s. | n.s. | **** | **** | **** | **** | **1.00** | 0.76 | 1.31 | **1.61** | 1.24 | 2.10 | **8** | **0.92** | 0.68 | 1.24 | **1.46** | 1.04 | 2.06 | **7** |
|  | IPI00975939.1 IPI00975939.1  IPI00019399.2 | **SAA2-SAA4 SAA2-SAA2 protein** | n.s. | n.s. | ** | t | >0.05 | >0.05 | **1.00** | 0.98 | 1.02 | **1.22** | 1.15 | 1.30 | **8** | **0.88** | 0.78 | 1.00 | **1.08** | 0.99 | 1.18 | **7** |
|  | IPI00022391.1 | APCS Serum amyloid P-component | n.s. | n.s. | n.s. | ** | >0.05 | >0.05 | **1.00** | 0.79 | 1.27 | **1.20** | 0.94 | 1.53 | **6** | **1.68** | 1.15 | 2.44 | **1.89** | 1.19 | 2.99 | **5** |
| **Inflammatory response** | | | | | | | | | | | | | | | | | | | | | | |
|  | IPI00027235.1 IPI00939169.1 IPI00162735.3 | ATRN Attractin | n.s. | n.s. | t | **** | >0.05 | >0.05 | **1.00** | 0.90 | 1.11 | **0.91** | 0.82 | 1.01 | **8** | **0.98** | 0.87 | 1.11 | **0.96** | 0.86 | 1.08 | **7** |
|  | IPI00010295.1 | CPN1 Carboxypeptidase N catalytic chain | n.s. | n.s. | n.s. | ** | >0.05 | >0.05 | **1.00** | 0.92 | 1.09 | **1.01** | 0.93 | 1.11 | **8** | **0.82** | 0.74 | 0.90 | **0.96** | 0.92 | 1.00 | **7** |
|  | IPI00479116.2 | CPN2 Carboxypeptidase N subunit 2 | n.s. | n.s. | n.s. | **** | >0.05 | >0.05 | **1.00** | 0.91 | 1.09 | **1.03** | 0.93 | 1.14 | **8** | **0.88** | 0.77 | 1.02 | **0.99** | 0.85 | 1.15 | **7** |
| **Immunity** | | | | | | | | | | | | | | | | | | | | | | |
|  | IPI00293925.2 IPI00419744.4 IPI00293925.2 | **FCN3 Ficolin-3** | n.s. | n.s. | * | **** | >0.05 | >0.05 | **1.00** | 0.44 | 2.25 | **0.84** | 0.40 | 1.80 | **6** | **1.29** | 0.49 | 3.39 | **1.19** | 0.47 | 2.98 | **6** |
|  | IPI00027547.2 IPI00847793.1 | DCD Dermcidin | n.s. | n.s. | n.s. | ** | >0.05 | >0.05 | **1.00** | 0.86 | 1.17 | **0.96** | 0.76 | 1.21 | **8** | **0.99** | 0.81 | 1.20 | **1.13** | 0.85 | 1.50 | **7** |
|  | IPI00719373.3 IPI00972963.1 IPI00827875.1 | IGLC1 IGL@ protein | n.s. | n.s. | n.s. | **** | >0.05 | >0.05 | **1.00** | 0.72 | 1.38 | **0.92** | 0.60 | 1.41 | **6** | **1.07** | 0.73 | 1.57 | **1.04** | 0.73 | 1.48 | **5** |
|  | IPI00163207.1 IPI00394992.1 | **PGLYRP2 Isoform 1 of N-acetylmuramoyl-L-alanine amidase** | n.s. | n.s. | *** | **** | >0.05 | ** | **1.00** | 0.88 | 1.14 | **0.89** | 0.77 | 1.04 | **8** | **1.09** | 0.96 | 1.23 | **0.85** | 0.73 | 0.98 | **7** |
| **Complement factors** | | | | | | | | | | | | | | | | | | | | | | |
|  | IPI00009793.4 IPI00872573.4 | **C1RL Complement C1r subcomponent-like protein** | n.s. | n.s. | * | *** | >0.05 | >0.05 | **1.00** | 0.91 | 1.09 | **1.15** | 1.02 | 1.30 | **8** | **0.82** | 0.68 | 0.98 | **0.98** | 0.83 | 1.17 | **7** |
|  | IPI00017696.1 IPI00749179.2 IPI00017696.1 | **C1S Complement C1s subcomponent** | n.s. | n.s. | * | ** | >0.05 | >0.05 | **1.00** | 0.93 | 1.08 | **1.16** | 1.08 | 1.24 | **8** | **1.02** | 0.92 | 1.14 | **1.11** | 1.02 | 1.20 | **7** |
|  | IPI00303963.1 IPI00645500.5 | C2 Complement C2 (Fragment) | n.s. | n.s. | n.s. | **** | >0.05 | >0.05 | **1.00** | 0.84 | 1.19 | **0.91** | 0.78 | 1.06 | **8** | **1.26** | 1.03 | 1.55 | **1.09** | 0.91 | 1.31 | **7** |
|  | IPI00021727.1 | C4BPA C4b-binding protein alpha chain | n.s. | * | n.s. | ** | >0.05 | >0.05 | **1.00** | 0.86 | 1.17 | **1.19** | 1.10 | 1.28 | **8** | **1.96** | 1.49 | 2.59 | **2.34** | 1.74 | 3.15 | **7** |
|  | IPI00032291.2 | **C5 Complement C5** | n.s. | n.s. | * | ** | >0.05 | * | **1.00** | 0.92 | 1.08 | **1.07** | 0.99 | 1.15 | **8** | **1.00** | 0.91 | 1.09 | **1.21** | 1.11 | 1.31 | **7** |
|  | IPI00879709.3 | **C6 Complement component C6 precursor** | n.s. | n.s. | ** | *** | >0.05 | >0.05 | **1.00** | 0.90 | 1.11 | **1.12** | 1.02 | 1.24 | **8** | **1.05** | 0.98 | 1.13 | **1.24** | 1.16 | 1.32 | **7** |
|  | IPI00296608.6 | C7 Complement component C7 | n.s. | n.s. | n.s. | **** | >0.05 | >0.05 | **1.00** | 0.80 | 1.25 | **1.13** | 0.90 | 1.42 | **8** | **1.01** | 0.76 | 1.33 | **0.99** | 0.78 | 1.26 | **7** |
|  | IPI00011252.1 | **C8A Complement component C8 alpha chain** | n.s. | n.s. | * | *** | >0.05 | * | **1.00** | 0.91 | 1.10 | **1.06** | 0.97 | 1.16 | **8** | **1.03** | 0.94 | 1.12 | **1.24** | 1.11 | 1.39 | **7** |
|  | IPI00294395.1 | C8B Complement component C8 beta chain | n.s. | n.s. | n.s. | n.s. | >0.05 | >0.05 | **1.00** | 0.95 | 1.06 | **1.03** | 0.98 | 1.08 | **8** | **0.97** | 0.87 | 1.08 | **1.01** | 0.94 | 1.08 | **7** |
|  | IPI00011261.2 | C8G Complement component C8 gamma chain | n.s. | n.s. | n.s. | n.s. | >0.05 | >0.05 | **1.00** | 0.95 | 1.05 | **1.07** | 1.01 | 1.13 | **8** | **0.92** | 0.79 | 1.07 | **1.09** | 1.03 | 1.14 | **7** |
|  | IPI00022395.1 | **C9 Complement component C9** | n.s. | n.s. | **** | * | *** | *** | **1.00** | 0.92 | 1.09 | **1.43** | 1.34 | 1.51 | **8** | **0.90** | 0.80 | 1.00 | **1.42** | 1.36 | 1.49 | **7** |
|  | IPI00019591.2 | **CFB cDNA FLJ55673, highly similar to Complement factor B** | t | n.s. | * | *** | >0.05 | * | **1.00** | 0.93 | 1.08 | **1.04** | 0.97 | 1.12 | **8** | **0.98** | 0.91 | 1.07 | **1.17** | 1.07 | 1.29 | **7** |
|  | IPI00165972.3 | CFD Complement factor D preproprotein | * | n.s. | n.s. | *** | * | >0.05 | **1.00** | 0.87 | 1.15 | **0.80** | 0.70 | 0.91 | **8** | **0.83** | 0.71 | 0.98 | **0.89** | 0.80 | 0.99 | **7** |
|  | IPI00029739.5 | CFH Isoform 1 of Complement factor H | * | t | n.s. | ** | >0.05 | * | **1.00** | 0.97 | 1.03 | **0.97** | 0.92 | 1.03 | **8** | **1.06** | 1.01 | 1.11 | **1.17** | 1.10 | 1.25 | **7** |
|  | IPI00011264.2 | CFHR1 Complement factor H-related protein 1 | n.s. | n.s. | n.s. | **** | >0.05 | >0.05 | **1.00** | 0.79 | 1.27 | **0.99** | 0.72 | 1.36 | **8** | **1.19** | 1.03 | 1.37 | **1.11** | 0.92 | 1.34 | **7** |
|  | IPI00291867.4 IPI00796990.4 IPI00796990.4 IPI00291867.4 | CFI Complement factor I | n.s. | n.s. | n.s. | * | >0.05 | >0.05 | **1.00** | 0.94 | 1.06 | **1.05** | 0.95 | 1.16 | **8** | **1.00** | 0.92 | 1.10 | **1.05** | 0.93 | 1.19 | **7** |
|  | IPI00877698.2 IPI00291866.5 IPI00877698.2 IPI00556459.2 IPI00291866.5 | **SERPING1 cDNA FLJ58826, highly similar to Plasma protease C1 inhibitor; SERPING1 Plasma protease C1 inhibitor** | n.s. | n.s. | ** | *** | >0.05 | >0.05 | **1.00** | 0.92 | 1.09 | **1.15** | 1.08 | 1.23 | **8** | **0.92** | 0.83 | 1.01 | **1.06** | 0.95 | 1.17 | **7** |
|  | IPI00021364.1 | CFP Properdin / Complement factor P | t | n.s. | t | **** | * | >0.05 | **1.00** | 0.83 | 1.20 | **0.81** | 0.68 | 0.97 | **8** | **0.92** | 0.79 | 1.07 | **0.94** | 0.77 | 1.13 | **7** |
|  | IPI00400826.1 IPI00954954.1 IPI00400826.1 IPI00976752.2 IPI00954954.1 IPI00400826.1 IPI00291262.3 | CLU Clusterin / Apolipoprotein J | n.s. | n.s. | n.s. | t | >0.05 | >0.05 | **1.00** | 0.91 | 1.10 | **1.05** | 0.95 | 1.17 | **8** | **1.07** | 1.01 | 1.14 | **1.21** | 1.12 | 1.30 | **7** |
| **Clotting/coagulation** | | | | | | | | | | | | | | | | | | | | | | |
|  | IPI00019576.1 | **F10 Coagulation factor X** | n.s. | n.s. | * | **** | * | >0.05 | **1.00** | 0.87 | 1.14 | **0.83** | 0.71 | 0.96 | **8** | **1.07** | 0.87 | 1.32 | **0.98** | 0.78 | 1.23 | **7** |
|  | IPI00019581.2 | **F12 Coagulation factor XII** | n.s. | n.s. | ** | **** | * | >0.05 | **1.00** | 0.87 | 1.15 | **0.88** | 0.75 | 1.03 | **8** | **0.96** | 0.84 | 1.10 | **0.87** | 0.76 | 1.00 | **7** |
|  | IPI00966520.1 IPI00654888.4 | **KLKB1 Plasma kallikrein** | n.s. | n.s. | * | ** | >0.05 | >0.05 | **1.00** | 0.94 | 1.06 | **0.88** | 0.81 | 0.95 | **8** | **1.00** | 0.91 | 1.10 | **0.95** | 0.86 | 1.04 | **7** |
|  | IPI00019568.1 | F2 Prothrombin (Fragment) | n.s. | n.s. | n.s. | *** | >0.05 | >0.05 | **1.00** | 0.95 | 1.06 | **0.92** | 0.86 | 0.98 | **8** | **0.95** | 0.88 | 1.01 | **0.96** | 0.90 | 1.02 | **7** |
|  | IPI00032179.3 | SERPINC1 Antithrombin-III | * | n.s. | n.s. | n.s. | >0.05 | >0.05 | **1.00** | 0.97 | 1.03 | **0.90** | 0.86 | 0.95 | **8** | **0.92** | 0.90 | 0.95 | **0.96** | 0.94 | 0.98 | **7** |
|  | IPI00879573.1 | **SERPIND1 Heparin cofactor 2** | n.s. | n.s. | *** | **** | *** | >0.05 | **1.00** | 0.90 | 1.11 | **0.74** | 0.67 | 0.82 | **8** | **1.18** | 1.04 | 1.35 | **1.03** | 0.89 | 1.19 | **7** |
|  | IPI00911093.1 IPI00940493.1 | PROC cDNA FLJ51034, highly similar to Vitamin K-dependent protein C | * | n.s. | n.s. | **** | >0.05 | >0.05 | **1.00** | 0.76 | 1.32 | **0.86** | 0.63 | 1.17 | **8** | **0.73** | 0.52 | 1.01 | **0.85** | 0.62 | 1.17 | **7** |
|  | IPI00294004.1 | PROS1 Vitamin K-dependent protein S | n.s. | n.s. | n.s. | *** | >0.05 | >0.05 | **1.00** | 0.95 | 1.05 | **1.02** | 0.95 | 1.09 | **8** | **1.22** | 1.09 | 1.35 | **1.15** | 0.98 | 1.36 | **7** |
|  | IPI00329775.8 | **CPB2 Isoform 1 of Carboxypeptidase B2** | n.s. | n.s. | * | **** | >0.05 | >0.05 | **1.00** | 0.87 | 1.15 | **0.88** | 0.75 | 1.02 | **8** | **1.19** | 1.00 | 1.41 | **1.06** | 0.88 | 1.27 | **7** |
|  | IPI01012492.1 IPI00215894.1 | KNG1 Isoform LMW of Kininogen-1 | * | n.s. | n.s. | ** | >0.05 | >0.05 | **1.00** | 0.95 | 1.06 | **0.87** | 0.81 | 0.94 | **6** | **0.84** | 0.77 | 0.92 | **0.93** | 0.79 | 1.09 | **5** |
| **Angiotensin etc** | | | | | | | | | | | | | | | | | | | | | | |
|  | IPI00032220.3 | AGT Angiotensinogen | n.s. | t | n.s. | n.s. | >0.05 | >0.05 | **1.00** | 0.95 | 1.06 | **0.98** | 0.92 | 1.04 | **8** | **0.86** | 0.79 | 0.93 | **0.85** | 0.80 | 0.91 | **7** |
| **Apolipoproteins** | | | | | | | | | | | | | | | | | | | | | | |
|  | IPI00021854.1 | APOA2 Apolipoprotein A-II | n.s. | n.s. | n.s. | **** | >0.05 | >0.05 | **1.00** | 0.88 | 1.13 | **0.93** | 0.82 | 1.05 | **8** | **0.94** | 0.79 | 1.13 | **0.87** | 0.72 | 1.05 | **7** |
|  | IPI00304273.2 | **APOA4 Apolipoprotein A-IV** | * | n.s. | **** | ** | **** | *** | **1.00** | 0.86 | 1.16 | **0.40** | 0.34 | 0.46 | **8** | **0.85** | 0.78 | 0.93 | **0.49** | 0.42 | 0.55 | **7** |
|  | IPI00021855.1 | APOC1 Apolipoprotein C-I | n.s. | n.s. | n.s. | ** | >0.05 | >0.05 | **1.00** | 0.81 | 1.23 | **1.04** | 0.83 | 1.30 | **8** | **0.89** | 0.69 | 1.14 | **0.67** | 0.58 | 0.78 | **7** |
|  | IPI00021856.3 | APOC2 Apolipoprotein C-II | n.s. | n.s. | n.s. | ** | >0.05 | >0.05 | **1.00** | 0.84 | 1.18 | **1.45** | 1.20 | 1.75 | **4** | **1.32** | 0.77 | 2.24 | **1.29** | 0.87 | 1.92 | **3** |
|  | IPI00657670.1 IPI00657670.1 IPI00021857.1 | APOC3 Apolipoprotein C-III variant 1 | n.s. | n.s. | n.s. | ** | >0.05 | >0.05 | **1.00** | 0.85 | 1.17 | **0.95** | 0.78 | 1.16 | **8** | **1.07** | 0.73 | 1.56 | **0.97** | 0.76 | 1.24 | **7** |
|  | IPI00021842.1 | APOE Apolipoprotein E | n.s. | n.s. | n.s. | t | >0.05 | >0.05 | **1.00** | 0.94 | 1.07 | **1.04** | 0.94 | 1.14 | **8** | **0.87** | 0.80 | 0.95 | **0.91** | 0.85 | 0.96 | **7** |
|  | IPI00298828.3 | APOH Beta-2-glycoprotein 1 | n.s. | n.s. | n.s. | *** | >0.05 | >0.05 | **1.00** | 0.96 | 1.04 | **0.94** | 0.88 | 1.02 | **8** | **0.95** | 0.83 | 1.08 | **0.93** | 0.82 | 1.05 | **7** |
| **Growth factors** | | | | | | | | | | | | | | | | | | | | | | |
|  | IPI00925635.1 IPI00020996.5 | **IGFALS Insulin-like growth factor-binding protein complex acid labile subunit** | n.s. | * | **** | ** | ** | ** | **1.00** | 0.95 | 1.05 | **0.81** | 0.75 | 0.86 | **8** | **0.81** | 0.76 | 0.87 | **0.63** | 0.57 | 0.69 | **7** |
|  | IPI00967086.1 IPI00029193.1 | HGFAC Hepatocyte growth factor activator | t | n.s. | t | **** | * | >0.05 | **1.00** | 0.90 | 1.11 | **0.86** | 0.75 | 0.99 | **8** | **0.71** | 0.62 | 0.82 | **0.72** | 0.63 | 0.83 | **7** |
| **Keratins** | | | | | | | | | | | | | | | | | | | | | | |
|  | IPI00220327.4 | KRT1 Keratin, type II cytoskeletal 1 | n.s. | n.s. | n.s. | n.s. | >0.05 | >0.05 | **1.00** | 0.82 | 1.21 | **1.53** | 1.17 | 2.01 | **8** | **1.43** | 1.12 | 1.83 | **1.80** | 1.32 | 2.46 | **7** |
| **Phospholipid biosynthesis , phosphatidylinositol and GPI anchor** | | | | | | | | | | | | | | | | | | | | | | |
|  | IPI00299503.2 | GPLD1 Isoform 1 of Phosphatidylinositol-glycan-specific phospholipase D | n.s. | n.s. | t | ** | >0.05 | >0.05 | **1.00** | 0.76 | 1.32 | **0.66** | 0.53 | 0.82 | **6** | **0.98** | 0.75 | 1.29 | **0.86** | 0.78 | 0.95 | **5** |
| **Lipid catabolic process** | | | | | | | | | | | | | | | | | | | | | | |
|  | IPI00166729.4 | AZGP1 Zinc-alpha-2-glycoprotein | n.s. | n.s. | n.s. | **** | >0.05 | >0.05 | **1.00** | 0.86 | 1.16 | **1.02** | 0.84 | 1.24 | **8** | **1.09** | 0.84 | 1.42 | **1.15** | 0.92 | 1.43 | **7** |
| **Proteases and peptidases** | | | | | | | | | | | | | | | | | | | | | | |
|  | IPI00064667.5 | **CNDP1 Beta-Ala-His dipeptidase (= Carnosine dipeptidase 1)** | n.s. | n.s. | **** | *** | ** | *** | **1.00** | 0.91 | 1.10 | **0.75** | 0.67 | 0.84 | **8** | **1.11** | 0.98 | 1.25 | **0.74** | 0.65 | 0.84 | **7** |
| **Misc protease inhibitors** | | | | | | | | | | | | | | | | | | | | | | |
|  | IPI00328609.3 | **SERPINA4 Kallistatin** | n.s. | * | *** | t | *** | * | **1.00** | 0.95 | 1.05 | **0.70** | 0.66 | 0.75 | **8** | **1.11** | 1.01 | 1.22 | **0.90** | 0.85 | 0.95 | **7** |
|  | IPI00027482.1 | SERPINA6 Corticosteroid-binding globulin | n.s. | n.s. | n.s. | ** | >0.05 | >0.05 | **1.00** | 0.91 | 1.10 | **0.86** | 0.76 | 0.99 | **8** | **0.94** | 0.86 | 1.04 | **0.98** | 0.90 | 1.07 | **7** |
|  | IPI00292946.1 | SERPINA7 Thyroxine-binding globulin | n.s. | n.s. | n.s. | *** | >0.05 | >0.05 | **1.00** | 0.89 | 1.12 | **0.94** | 0.83 | 1.08 | **8** | **0.91** | 0.81 | 1.03 | **0.92** | 0.77 | 1.09 | **7** |
|  | IPI00006114.5 | SERPINF1 Pigment epithelium-derived factor | n.s. | n.s. | n.s. | ** | >0.05 | >0.05 | **1.00** | 0.92 | 1.08 | **0.88** | 0.79 | 0.97 | **8** | **1.00** | 0.89 | 1.12 | **0.95** | 0.87 | 1.03 | **7** |
|  | IPI00879231.1 | SERPINF2 Alpha-2-antiplasmin | * | n.s. | n.s. | *** | >0.05 | >0.05 | **1.00** | 0.94 | 1.07 | **0.92** | 0.86 | 0.99 | **8** | **0.91** | 0.85 | 0.97 | **0.99** | 0.93 | 1.04 | **7** |
|  | IPI00292530.1 | ITIH1 Inter-alpha-trypsin inhibitor heavy chain H1 | n.s. | n.s. | n.s. | ** | >0.05 | >0.05 | **1.00** | 0.95 | 1.05 | **0.91** | 0.86 | 0.97 | **8** | **1.06** | 0.98 | 1.16 | **1.06** | 0.95 | 1.19 | **7** |
|  | IPI00645038.1 IPI00305461.4 | **ITIH2 Uncharacterized protein** | t | n.s. | ** | ** | ** | >0.05 | **1.00** | 0.94 | 1.06 | **0.81** | 0.74 | 0.89 | **8** | **1.05** | 1.01 | 1.08 | **0.97** | 0.91 | 1.04 | **7** |
|  | IPI00876950.1 IPI00953573.2 | **ITIH3 Isoform 2 of Inter-alpha-trypsin inhibitor heavy chain H3** | n.s. | n.s. | ** | **** | ** | >0.05 | **1.00** | 0.88 | 1.14 | **1.33** | 1.16 | 1.54 | **8** | **1.15** | 1.00 | 1.32 | **1.29** | 1.09 | 1.53 | **7** |
|  | IPI00896419.3 IPI00944960.1 IPI00896419.3 IPI00944960.1 IPI00896419.3 IPI00896413.1 IPI00218192.3 | **ITIH4 Isoform 1 of Inter-alpha-trypsin inhibitor heavy chain H4** | t | n.s. | ** | *** | >0.05 | ** | **1.00** | 0.93 | 1.07 | **1.04** | 0.98 | 1.10 | **8** | **0.97** | 0.90 | 1.04 | **1.14** | 1.08 | 1.20 | **7** |
|  | IPI00022426.1 | AMBP Protein AMBP | n.s. | n.s. | n.s. | t | >0.05 | >0.05 | **1.00** | 0.90 | 1.12 | **1.03** | 0.93 | 1.15 | **8** | **0.86** | 0.83 | 0.88 | **0.97** | 0.91 | 1.03 | **7** |
|  | IPI00025426.3 | PZP Isoform 1 of Pregnancy zone protein | * | n.s. | n.s. | ** | >0.05 | * | **1.00** | 0.68 | 1.48 | **1.23** | 0.82 | 1.85 | **4** | **1.28** | 0.51 | 3.21 | **0.59** | 0.24 | 1.44 | **3** |
| **Actin binding** | | | | | | | | | | | | | | | | | | | | | | |
|  | IPI00026314.1 | **GSN Isoform 1 of Gelsolin** | n.s. | n.s. | **** | ** | *** | ** | **1.00** | 0.94 | 1.07 | **0.74** | 0.68 | 0.80 | **8** | **0.89** | 0.81 | 0.98 | **0.69** | 0.68 | 0.70 | **7** |
| **Transport** | | | | | | | | | | | | | | | | | | | | | | |
|  | IPI00953689.1 IPI00953689.1 IPI00022431.2 | **AHSG Alpha-2-HS-glycoprotein (= Fetuin-A)** | n.s. | n.s. | * | ** | * | >0.05 | **1.00** | 0.91 | 1.10 | **0.75** | 0.67 | 0.85 | **8** | **0.96** | 0.82 | 1.13 | **0.87** | 0.75 | 1.00 | **7** |
|  | IPI00968027.1 IPI00555812.5 IPI00965085.1 | GC vitamin D-binding protein | * | n.s. | n.s. | **** | >0.05 | >0.05 | **1.00** | 0.95 | 1.06 | **0.98** | 0.92 | 1.04 | **8** | **0.93** | 0.86 | 1.01 | **0.98** | 0.91 | 1.05 | **7** |
|  | IPI00019943.1 | **AFM Afamin** | n.s. | n.s. | **** | ** | *** | ** | **1.00** | 0.94 | 1.07 | **0.76** | 0.70 | 0.82 | **8** | **1.05** | 0.97 | 1.14 | **0.80** | 0.74 | 0.88 | **7** |
|  | IPI00022488.1 | HPX Hemopexin | n.s. | n.s. | n.s. | **** | >0.05 | >0.05 | **1.00** | 0.91 | 1.10 | **0.92** | 0.83 | 1.02 | **8** | **1.00** | 0.92 | 1.09 | **0.99** | 0.91 | 1.07 | **7** |
|  | IPI00844536.2 IPI00480192.1 IPI00022420.3 | **RBP4 Uncharacterized protein** | n.s. | n.s. | ** | **** | ** | >0.05 | **1.00** | 0.87 | 1.14 | **0.72** | 0.63 | 0.83 | **8** | **1.02** | 0.85 | 1.22 | **0.90** | 0.75 | 1.09 | **7** |
| **Adhesion** | | | | | | | | | | | | | | | | | | | | | | |
|  | IPI00922213.2 | FN1 cDNA FLJ53292, highly similar to Homo sapiens fibronectin 1 (FN1), transcript variant 5, mRNA | n.s. | n.s. | n.s. | t | >0.05 | >0.05 | **1.00** | 0.70 | 1.42 | **1.57** | 0.88 | 2.81 | **6** | **1.60** | 0.88 | 2.93 | **1.89** | 1.20 | 2.97 | **6** |
|  | IPI00867588.2 IPI00855785.2 IPI00855777.2 IPI00845263.3 IPI00479723.5 IPI00414283.7 IPI00339228.2 IPI00339227.5 IPI00339223.2 IPI00022418.2 IPI00867588.2 IPI00855785.2 IPI00479723.5 IPI00339228.2 IPI00022418.2 IPI00855785.2 IPI00845263.3 IPI00339227.5 IPI00339226.4 IPI00339223.2 IPI00022418.2 | FN1 Fibronectin | n.s. | n.s. | n.s. | ** | >0.05 | >0.05 | **1.00** | 0.76 | 1.32 | **1.17** | 0.78 | 1.76 | **8** | **1.51** | 0.99 | 2.30 | **1.24** | 0.84 | 1.83 | **7** |
|  | IPI00298971.1 | VTN Vitronectin | n.s. | n.s. | t | ** | >0.05 | >0.05 | **1.00** | 0.95 | 1.06 | **1.06** | 1.00 | 1.13 | **8** | **1.11** | 1.04 | 1.19 | **1.21** | 1.13 | 1.29 | **7** |
|  | IPI00827893.1 IPI00984539.2 IPI00956122.2 IPI00828192.2 IPI00828064.2 IPI00827555.2 IPI00418465.5 IPI00305064.2 IPI00984539.2 IPI00956122.2 IPI00827650.2 IPI00418465.5 IPI00305064.2 | CD44 CD44 antigen | n.s. | n.s. | n.s. | **** | >0.05 | >0.05 | **1.00** | 0.72 | 1.38 | **1.00** | 0.74 | 1.36 | **6** | **0.97** | 0.75 | 1.25 | **1.03** | 0.74 | 1.42 | **5** |
|  | IPI00296534.2 IPI00218803.3 | FBLN1 Fibulin-1 | n.s. | n.s. | n.s. | t | >0.05 | >0.05 | **1.00** | 0.90 | 1.11 | **1.15** | 1.04 | 1.27 | **8** | **0.84** | 0.73 | 0.97 | **0.87** | 0.76 | 1.01 | **7** |
|  | IPI00023673.1 IPI01010990.1 IPI00023673.1 | LGALS3BP Galectin-3-binding protein | n.s. | n.s. | n.s. | ** | >0.05 | >0.05 | **1.00** | 0.93 | 1.08 | **1.18** | 1.00 | 1.39 | **6** | **0.83** | 0.64 | 1.09 | **0.87** | 0.57 | 1.34 | **5** |
| **Miscellanous** | | | | | | | | | | | | | | | | | | | | | | |
|  | IPI00009028.2 IPI00792115.1 IPI00009028.2 | **CLEC3B Tetranectin** | n.s. | n.s. | * | * | * | >0.05 | **1.00** | 0.91 | 1.10 | **0.80** | 0.75 | 0.85 | **6** | **0.90** | 0.80 | 1.02 | **0.82** | 0.76 | 0.89 | **5** |
|  | IPI00645849.1 IPI00003351.2 | ECM1 Extracellular matrix protein 1 | n.s. | n.s. | t | *** | >0.05 | >0.05 | **1.00** | 0.88 | 1.14 | **0.84** | 0.69 | 1.01 | **8** | **0.84** | 0.67 | 1.04 | **0.72** | 0.58 | 0.89 | **7** |
|  | IPI00746623.2 IPI00964994.1 IPI00746623.2 | HABP2 Hyaluronan-binding protein 2 | * | n.s. | n.s. | **** | >0.05 | >0.05 | **1.00** | 0.80 | 1.25 | **0.94** | 0.76 | 1.16 | **8** | **0.97** | 0.76 | 1.25 | **1.11** | 0.86 | 1.44 | **7** |
|  | IPI00022371.1 | **HRG Histidine-rich glycoprotein** | n.s. | n.s. | * | **** | >0.05 | >0.05 | **1.00** | 0.88 | 1.14 | **0.88** | 0.77 | 1.00 | **8** | **1.02** | 0.93 | 1.12 | **0.93** | 0.84 | 1.03 | **7** |
|  | IPI00020986.2 | LUM Lumican | n.s. | t | t | * | >0.05 | >0.05 | **1.00** | 0.93 | 1.08 | **0.80** | 0.72 | 0.90 | **8** | **0.75** | 0.70 | 0.80 | **0.70** | 0.64 | 0.77 | **7** |
|  | IPI00022445.1 | PPBP Platelet basic protein | t | n.s. | t | t | >0.05 | >0.05 | **1.00** | 0.77 | 1.30 | **1.03** | 0.81 | 1.31 | **6** | **1.41** | 1.26 | 1.57 | **0.65** | 0.42 | 1.01 | **5** |
| **Unknown function** | | | | | | | | | | | | | | | | | | | | | | |
|  | IPI00022417.4 IPI01012772.1 IPI00022417.4 | **LRG1 Leucine-rich alpha-2-glycoprotein** | n.s. | t | **** | ** | *** | ** | **1.00** | 0.91 | 1.10 | **1.70** | 1.51 | 1.91 | **8** | **0.80** | 0.70 | 0.91 | **1.23** | 1.12 | 1.34 | **7** |
|  | IPI00022895.8 | A1BG Isoform 1 of Alpha-1B-glycoprotein | n.s. | n.s. | n.s. | * | >0.05 | >0.05 | **1.00** | 0.93 | 1.07 | **1.00** | 0.93 | 1.08 | **8** | **1.07** | 1.00 | 1.15 | **1.07** | 0.97 | 1.19 | **7** |
