# Supplementary material for: Lower Fetuin-A, Retinol Binding Protein 4 and Several Metabolites after Gastric Bypass Compared to Sleeve Gastrectomy in Patients with Type 2 Diabetes
Source: PLoS One. 2014 May 6;9(5):e96489. doi: 10.1371/journal.pone.0096489 (PMC4011803; doi:10.1371/journal.pone.0096489)
Supplement: Table S2 — Summary statistics for all identified metabolites. Abundance (relative to averaged pre-operative GBP samples), S.E.M. and outcome of statistical analysis (Two-Way ANOVA followed by Sidak's post-test) are shown for all 45 metabolites identified. Abbreviations: *, p<0.05; **, p<0.01; ***, p<0.001, ****, p<0.0001; n.s., p>0.05. (DOCX) [file pone.0096489.s002.docx]

**Table S2**

**Summary statistics for all identified metabolites.** Abundance (relative to averaged pre-operative GBP samples), S.E.M. and outcome of statistical analysis (Two-Way ANOVA followed by Sidak’s post-test) are shown for all 45 metabolites identified. Abbreviations: *, p<0.05; **, p<0.01; ***, p<0.001, ****, p<0.0001; n.s., p>0.05.

|  | **Pre GBP** | | **Post GBP** | | **Pre SG** | | **Post SG** | | **Effect of surgery** | **Sidak's post-test** | |
| --- | --- | --- | --- | --- | --- | --- | --- | --- | --- | --- | --- |
| **Metabolite** | Mean | SEM | Mean | SEM | Mean | SEM | Mean | SEM |  | **post Vs pre GBP** | **post Vs pre SG** |
| 2-Hydroxybutyric acid | **1.00** | 0.14 | **1.16** | 0.17 | **0.97** | 0.12 | **2.01** | 0.39 | * | n.s. | * |
| 3-Methyl-2-oxopentanoic acid | **1.00** | 0.11 | **0.93** | 0.14 | **0.81** | 0.12 | **1.57** | 0.24 | * | n.s. | ** |
| Alanine | **1.00** | 0.09 | **0.52** | 0.04 | **1.27** | 0.19 | **0.68** | 0.06 | *** | * | ** |
| Arachidonic acid | **1.00** | 0.13 | **1.17** | 0.14 | **0.78** | 0.13 | **0.74** | 0.11 | n.s. | n.s. | n.s. |
| Behenic acid | **1.00** | 0.13 | **1.04** | 0.13 | **0.79** | 0.26 | **0.53** | 0.13 | n.s. | n.s. | n.s. |
| cis-11,14-Eicosadienoic acid | **1.00** | 0.12 | **1.01** | 0.16 | **0.80** | 0.09 | **0.62** | 0.05 | n.s. | n.s. | n.s. |
| cis-4,7,10,13,16,19-Docosahexaenoic acid | **1.00** | 0.11 | **1.23** | 0.17 | **0.86** | 0.14 | **0.79** | 0.07 | n.s. | n.s. | n.s. |
| cis-8,11,14-Eicosatrienoic acid | **1.00** | 0.22 | **1.03** | 0.17 | **0.94** | 0.15 | **0.66** | 0.07 | n.s. | n.s. | n.s. |
| cis-Aconitic acid | **1.00** | 0.10 | **0.78** | 0.07 | **1.16** | 0.16 | **1.10** | 0.17 | n.s. | n.s. | n.s. |
| Citric acid | **1.00** | 0.10 | **0.76** | 0.07 | **1.00** | 0.07 | **0.88** | 0.07 | ** | * | n.s. |
| Cysteine | **1.00** | 0.10 | **0.99** | 0.10 | **0.90** | 0.10 | **0.86** | 0.10 | n.s. | n.s. | n.s. |
| Decanoic acid | **1.00** | 0.12 | **0.67** | 0.08 | **0.86** | 0.07 | **0.77** | 0.07 | * | * | n.s. |
| Dodecanoic (Lauric) acid | **1.00** | 0.09 | **0.86** | 0.24 | **0.84** | 0.09 | **0.79** | 0.08 | n.s. | n.s. | n.s. |
| EDTA | **1.00** | 0.09 | **1.10** | 0.06 | **0.99** | 0.06 | **0.93** | 0.14 | n.s. | n.s. | n.s. |
| gamma-Linolenic acid | **1.00** | 0.09 | **0.79** | 0.14 | **0.62** | 0.10 | **0.54** | 0.08 | n.s. | n.s. | n.s. |
| Glutamic acid | **1.00** | 0.12 | **1.13** | 0.19 | **1.32** | 0.15 | **1.42** | 0.26 | n.s. | n.s. | n.s. |
| Glycine | **0.75** | 0.17 | **0.34** | 0.17 | **0.52** | 0.17 | **0.40** | 0.16 | n.s. | n.s. | n.s. |
| Heptadecanoic (Margaric) acid | **1.00** | 0.20 | **1.12** | 0.17 | **0.86** | 0.16 | **0.61** | 0.11 | n.s. | n.s. | n.s. |
| Histidine | **1.00** | 0.12 | **0.72** | 0.07 | **0.78** | 0.10 | **0.68** | 0.07 | * | * | n.s. |
| Isoleucine | **1.00** | 0.14 | **0.88** | 0.08 | **0.84** | 0.05 | **1.11** | 0.12 | n.s. | n.s. | n.s. |
| Lactic acid | **1.00** | 0.16 | **0.80** | 0.17 | **1.54** | 0.41 | **1.07** | 0.24 | n.s. | n.s. | n.s. |
| Leucine | **1.00** | 0.14 | **0.87** | 0.09 | **0.80** | 0.05 | **1.08** | 0.10 | n.s. | n.s. | n.s. |
| Lignoceric acid | **1.00** | 0.16 | **0.83** | 0.19 | **0.73** | 0.29 | **0.46** | 0.16 | n.s. | n.s. | n.s. |
| Linoleic acid | **1.00** | 0.08 | **0.78** | 0.13 | **0.74** | 0.13 | **0.67** | 0.09 | n.s. | n.s. | n.s. |
| L-Ornithine | **1.00** | 0.16 | **0.88** | 0.12 | **1.04** | 0.15 | **1.09** | 0.14 | n.s. | n.s. | n.s. |
| Lysine | **1.00** | 0.09 | **0.82** | 0.10 | **0.93** | 0.18 | **0.95** | 0.18 | n.s. | n.s. | n.s. |
| Methionine | **1.00** | 0.17 | **0.76** | 0.08 | **0.75** | 0.04 | **0.84** | 0.08 | n.s. | n.s. | n.s. |
| Myristic acid | **1.00** | 0.17 | **0.76** | 0.14 | **0.94** | 0.14 | **0.85** | 0.14 | n.s. | n.s. | n.s. |
| N2-Acetyl-L-lysine | **1.00** | 0.08 | **1.02** | 0.12 | **0.89** | 0.10 | **0.88** | 0.11 | n.s. | n.s. | n.s. |
| Octadecanoic (Stearic) acid | **1.00** | 0.11 | **1.00** | 0.10 | **0.78** | 0.14 | **0.66** | 0.10 | n.s. | n.s. | n.s. |
| Oleic acid | **1.00** | 0.09 | **1.00** | 0.10 | **0.85** | 0.09 | **0.88** | 0.08 | n.s. | n.s. | n.s. |
| Palmitelaidic acid | **1.00** | 0.15 | **0.93** | 0.22 | **1.01** | 0.22 | **0.90** | 0.27 | n.s. | n.s. | n.s. |
| Palmitic acid | **1.00** | 0.07 | **1.03** | 0.09 | **0.78** | 0.11 | **0.77** | 0.09 | n.s. | n.s. | n.s. |
| Palmitoleic acid | **0.88** | 0.19 | **0.70** | 0.29 | **1.11** | 0.19 | **0.77** | 0.21 | n.s. | n.s. | n.s. |
| Pentadecanoic acid | **1.00** | 0.14 | **0.88** | 0.10 | **0.96** | 0.13 | **0.85** | 0.13 | n.s. | n.s. | n.s. |
| Phenylalanine | **1.00** | 0.14 | **0.84** | 0.05 | **0.81** | 0.04 | **0.85** | 0.05 | n.s. | n.s. | n.s. |
| Proline | **1.00** | 0.12 | **0.56** | 0.09 | **1.00** | 0.18 | **0.71** | 0.11 | ** | * | n.s. |
| p-Toluic acid | **1.00** | 0.06 | **1.00** | 0.08 | **0.95** | 0.08 | **0.89** | 0.07 | n.s. | n.s. | n.s. |
| Quinic acid | **1.00** | 0.07 | **0.92** | 0.08 | **0.96** | 0.10 | **0.95** | 0.08 | n.s. | n.s. | n.s. |
| Serine | **1.00** | 0.12 | **0.90** | 0.09 | **0.94** | 0.13 | **0.92** | 0.10 | n.s. | n.s. | n.s. |
| Threonine | **1.00** | 0.16 | **0.84** | 0.10 | **0.80** | 0.14 | **0.78** | 0.08 | * | n.s. | n.s. |
| trans-9-Heptadecenoic acid | **1.00** | 0.13 | **0.91** | 0.16 | **0.92** | 0.13 | **0.87** | 0.14 | n.s. | n.s. | n.s. |
| Tryptophan | **1.00** | 0.14 | **0.74** | 0.07 | **0.75** | 0.10 | **0.72** | 0.18 | n.s. | n.s. | n.s. |
| Tyrosine | **1.00** | 0.16 | **0.72** | 0.07 | **0.72** | 0.08 | **0.80** | 0.16 | n.s. | n.s. | n.s. |
| Valine | **1.00** | 0.08 | **0.89** | 0.08 | **0.96** | 0.07 | **1.10** | 0.10 | n.s. | n.s. | n.s. |
