# Supplementary material for: Lower Fetuin-A, Retinol Binding Protein 4 and Several Metabolites after Gastric Bypass Compared to Sleeve Gastrectomy in Patients with Type 2 Diabetes
Source: PLoS One. 2014 May 6;9(5):e96489. doi: 10.1371/journal.pone.0096489 (PMC4011803; doi:10.1371/journal.pone.0096489)
Supplement: Protocol S1 — Proteomic sub-study protocol detailing patient recruitment, exclusion criteria and study protocol. (DOCX) [file pone.0096489.s004.docx]

**Aims and objectives (main study):**

1. To study the changes of gut hormones during the early post-operative phase (<3 days) in patients undergoing either gastric bypass surgery or gastric sleeve resections

2. To study the effect of bariatric surgery (gastric bypass or gastric sleeve resection on glycaemia using continuous glucose monitoring system in the peri-operative phase.

**Proteomic sub-study inclusion protocol**

Patient recruitment:

Inclusion criteria:

1. patients with type 2 diabetes treated with metformin monotherapy planned to have gastric bypass surgery
2. patients with type 2 diabetes treated with metformin monotherapy planned to have gastric sleeve resection

Exclusion criteria:

1. Age <18, or >70
2. BMI < 35 kg/m2
3. Cardiovascular conditions including significant coronary artery disease, congestive heart failure
4. Severe pulmonary disease
5. Previous bariatric/gastric/small bowel operation.
6. Underlying endocrinology disorder or genetic syndrome related to obesity and secondary diabetes.
7. Steroid dependence
8. Exenatide treatment within 3 months of surgery
9. Previous surgery to pancreas, or other pancreatic pathology
10. Pregnant women

Study protocol: gut hormone and metabolic changes after bariatric surgery

- 1. 10 patients with type 2 diabetes selected to have laparoscopic gastric bypass surgery and 10 patients with type 2 diabetes selected to have laparoscopic gastric sleeve resection will be recruited as cases; and 10 patients considering these types of surgery will be recruited to take part as controls in the study.
     - Control patients will follow a diet to match that of patients undergoing surgery with the same duration of fasting and caloric intake.
  2. Demographics and diabetes history to be obtained.
  3. Standard oGTT will be performed 3 days prior to surgery and then at day 3 post operatively.
     - fasting blood tests then 75g oral glucose load, before sampling at 30, 60, 90, 120 minutes for glucose, insulin, c-peptide, free fatty acids, glucagon, Ghrelin, and incretin hormones (intact GIP and GLP -1). Other metabolic laboratory assessment on blood samples may also be carried out.
  4. Statistical analysis will be performed to identify any significant differences after surgery, and between the three groups. Baseline characteristics will be compared to ensure no significant differences between the groups prior to surgery
